# The mismatch negativity as an index of cognitive abilities in adults with Down syndrome

**DOI:** 10.1093/cercor/bhad233

**Published:** 2023-07-03

**Authors:** Fedal Saini, Fabio Masina, Jasmine Wells, Richard Rosch, Sarah Hamburg, Carla Startin, André Strydom

**Affiliations:** Department of Forensic and Neurodevelopmental Sciences, Institute of Psychiatry, Psychology & Neuroscience, King’s College London, 16 De Crespigny Park, London SE5 8AB, UK; IRCCS San Camillo Hospital, Via Alberoni, 70, 30126 Lido VE, Italy; Department of Forensic and Neurodevelopmental Sciences, Institute of Psychiatry, Psychology & Neuroscience, King’s College London, 16 De Crespigny Park, London SE5 8AB, UK; Department of Clinical Neurophysiology, King’s College Hospital NHS Foundation Trust, Golden Jubilee, Denmark Hill, London SE5 9RS, UK; Wellcome Centre for Human Neuroimaging, UCL Queen Square Institute of Neurology, University College London, Queen Square, London WC1N 3AR, UK; Department of Forensic and Neurodevelopmental Sciences, Institute of Psychiatry, Psychology & Neuroscience, King’s College London, 16 De Crespigny Park, London SE5 8AB, UK; Division of Psychiatry, University College London, Maple House, 149 Tottenham Ct Rd, London W1T 7BN, UK; Department of Forensic and Neurodevelopmental Sciences, Institute of Psychiatry, Psychology & Neuroscience, King’s College London, 16 De Crespigny Park, London SE5 8AB, UK; Division of Psychiatry, University College London, Maple House, 149 Tottenham Ct Rd, London W1T 7BN, UK; School of Psychology, University of Roehampton, Grove House, Roehampton Lane, London, SW15 5PJ, UK; Department of Forensic and Neurodevelopmental Sciences, Institute of Psychiatry, Psychology & Neuroscience, King’s College London, 16 De Crespigny Park, London SE5 8AB, UK; Division of Psychiatry, University College London, Maple House, 149 Tottenham Ct Rd, London W1T 7BN, UK

**Keywords:** Alzheimer’s disease (AD), event-related potentials (ERPs), Trisomy 21

## Abstract

Down syndrome (DS) is associated with an ultra-high risk of developing Alzheimer’s disease (AD). Understanding variability in pre-AD cognitive abilities may help understand cognitive decline in this population. The mismatch negativity (MMN) is an event-related potential component reflecting the detection of deviant stimuli that is thought to represent underlying memory processes, with reduced MMN amplitudes being associated with cognitive decline. To further understand the MMN in adults with DS without AD, we explored the relationships between MMN, age, and cognitive abilities (memory, language, and attention) in 27 individuals (aged 17–51) using a passive auditory oddball task. Statistically significant MMN was present only in 18 individuals up to 41 years of age and the latency were longer than canonical parameters reported in the literature. Reduced MMN amplitude was associated with lower memory scores, while longer MMN latencies were associated with poorer memory, verbal abilities, and attention. Therefore, the MMN may represent a valuable index of cognitive abilities in DS. In combination with previous findings, we hypothesize that while MMN response and amplitude may be associated with AD-related memory loss, MMN latency may be associated with speech signal processing. Future studies may explore the potential impact of AD on MMN in people with DS.

## Introduction

Down syndrome (DS) is the most common genetic form of intellectual disability and is caused by an extra copy of chromosome 21 (Antonarakis et al. 2020). Most individuals with DS present with moderate to severe intellectual disability (Lott and Dierssen 2010) and a cognitive profile marked by impairments in language, memory, and executive functioning (see Grieco et al. 2015 for a review). From a neuroanatomical perspective, people with DS exhibit reductions in fronto-temporal and cerebellar volumes (Teipel et al. 2003; Teipel et al. 2004; Annus et al. 2017; Pujol et al. 2018), together with white matter structural atrophy throughout the main association fibers and the corpus callosum later in life (see Saini et al. 2022 for a review).

Adults with DS are at ultra-high risk of developing Alzheimer disease (AD), with a lifetime prevalence of 90% (McCarron et al. 2014; Zis and Strydom 2018). Such risk is commonly attributed to overexpression of the Aβ-amyloid precursor protein gene (*APP*), which leads to brain amyloid deposition in most individuals with DS by the age of forty (Davidson et al. 2018). However, these neuropathological changes begin several years before symptom onset (Sperling et al. 2013; Teipel et al. 2020). Therefore, early detection of AD in DS is essential to optimize clinical interventions and subsequently improve quality of life.

A possible biomarker for the detection of cognitive decline is represented by the mismatch negativity (MMN), an event-related potential (ERP) component that is thought to be an objective index of stimulus discrimination and memory (Garrido et al. 2009; Näätänen et al. 2011; Bartha-Doering et al. 2015). The MMN is characterized by a negative deflection that occurs approximately 100–250 ms after a detectable sensorial change (i.e., deviant stimulus), embedded within a train of repetitive and predictive sounds (i.e., frequent stimuli; Näätänen et al. 2007). The MMN is elicited, at least in part, by pre-attentive levels of cortical hierarchy, as it is observable even in the absence of directed attention to stimuli (Naatanen and Näätänen 1992). This feature makes the MMN particularly useful for the investigation of clinical populations such as those with DS, who may have difficulty with sustained attention tasks with complex instructions. Furthermore, the MMN is commonly used in clinical settings to index cognitive decline in several neuropsychiatric and neurodevelopmental conditions such as schizophrenia, bipolar disorder, autism spectrum disorder (ASD), and dyslexia (Näätänen et al. 2011; Näätänen et al. 2012; Näätänen et al. 2014).

The hypothesis that the MMN may be considered a biomarker for aberrant cognitive functioning has been repeatedly supported. A recent review showed that auditory sensory memory is impaired in AD, such that people with AD were unable to retain short-term auditory memory traces (Bartha-Doering et al. 2015). Pekkonen (2000) found the MMN amplitude decreased for patients with AD in response to deviant auditory stimuli separated by inter-stimulus intervals of 3s. Given that the MMN amplitude for shorter inter-stimulus intervals was not affected, the authors proposed that patients with AD were unable to maintain a sensory memory trace and were subsequently unable to discern the novel from familiar auditory stimuli. This finding suggests that auditory sensory memory traces decay faster in patients with AD compared to healthy controls. Furthermore, delays in the onset of MMN generation have also been observed in AD. In a study using high-density EEG-3D vector field tomography, Papadaniil et al. (2016) found that the MMN latency was longer in patients with AD compared to healthy elderly controls.

In the transition between typical aging and AD, mild cognitive impairment (MCI) represents a prodromal stage before the development of dementia (Portet et al. 2006). Similar to patients with AD, individuals with MCI show attenuation of MMN amplitude compared to healthy controls (Mowszowski et al. 2012). In addition, a recent study by Ji et al. (2015) found longer MMN latencies in patients with MCI as compared to age-matched controls, without significant differences in MMN amplitude. Overall, these studies suggest that a longer MMN latency may be a promising biomarker of cognitive decline.

Whilst loss of cognitive abilities is a common feature of DS, particularly during middle adulthood where AD neuropathology develops (Grieco et al. 2015; Startin et al. 2019), few studies have investigated the MMN in DS. Studies that have explored MMN responses in this population reported a reduced rate of MMN generation, amplitude attenuation, and increased latency (Diaz and Zurron 1995; Lalo et al. 2005; César et al. 2010; Arisi et al. 2012). Avancini et al. (2020) found no association between MMN parameters and subsequent development of dementia symptoms (as measured with the Cambridge Cognitive Examination for Older Adults with Down syndrome; CAMCOG) over a one-year period. However, no studies have directly investigated the potential relationship between MMN parameters and specific cognitive abilities in people with DS. Understanding these relationships prior to the development of dementia in people with DS may be important for the interpretation of findings in those with cognitive decline.

A further aspect that can be derived from the MMN concerns auditory short-term memory, for which intact functioning is essential for language acquisition and speech (Jarrold et al. 2002). Given that expressive language ability represents one of the most affected areas of cognition in people with DS (Grieco et al. 2015), the MMN may provide valuable insights about the mechanisms underlying language processing in this population.

Therefore, this study aims to characterize the relationship between MMN amplitude and latency and cognitive abilities in adults with DS, by including a broader age range than previous studies, and several domains of cognitive functioning. To do so, we investigated the MMN in a group of both younger and older adults with DS. We then tested the potential association between MMN and age, IQ, verbal abilities, memory, and executive functioning.

## Materials and methods

### Study design

This study is configured as a cross-sectional investigation. Participants undertook a cognitive assessment using the London Down Syndrome Consortium (LonDownS) cognitive test battery (Startin et al. 2016). Demographic and clinical data were obtained through caregivers’ interview. The assessment took place either in the participant’s home or within University testing facilities, depending on participants’ preference. An EEG recording session was then offered at the Institute of Child Health (ICH) in London. The length of time between the cognitive assessment and EEG was kept to the minimum, especially for older adults (aged 36 years and over) as cognitive decline between sessions was more likely (median = 63 days; see Supplementary Table 1 for full details).

### Ethical considerations

Ethical approval for the study was secured from the North Wales West Research Ethics Committee (13/WA/0194), which included approval for cognitive and EEG testing. Where individuals had capacity to consent for themselves, written informed consent was obtained and all study-related material was provided in easy-read format. Where individuals did not have capacity to consent for themselves, a consultee was asked to sign a form to indicate their decision regarding the individuals’ inclusion based on their knowledge of the individual and his/her wishes, in accordance with the England and Wales Mental Capacity Act 2005.

### Participants

Participants with DS were recruited across England and Wales (with a focus on Greater London and Southeast England) via local care homes, DS support groups and on an ad hoc basis through an established network with National Health Service (NHS) Trust sites. Individuals were only invited to attend the EEG session if they were deemed suitable for EEG testing (i.e., able to tolerate an EEG cap for up to one hour), a decision that was based on discussions with participants, parents, and/or carers. All participants were aged 16 or over and had a genetically confirmed trisomy 21 diagnosis. Participants with an acute physical or mental health condition were excluded, as were those who were noncompliant with experimental instructions. Finally, only participants without significant hearing loss were included in the study (PTA ≤ 70 dB HL; Prescott et al. 1999). Hearing loss was measured using the Whisper Hearing Test (Prescott et al. 1999) and the inclusion threshold was “conversational voice” (50–60 dB; Whisper Hearing Test scores for each participant included in the study are provided in Supplementary Table 1. In total, 47 participants aged 17–58 years (*M* = 33.2 years, SD = 11.6, 26 females) met the above criteria and were included in the study.

### Cognitive assessment

All participants underwent extensive neuropsychological examination using the LonDownS battery, which involves a series of tests assessing general abilities, memory, language, executive functioning, and motor skills (Startin et al. 2016). Cognitive task outcomes selected for use in this study were measures of language, memory, attention, and general cognitive abilities (see Table 1). These tasks are commonly used in populations with intellectual disability and have good test–retest reliability (Startin et al. 2016). Interviews with participants’ caregivers were carried out to obtain demographic, medical information (i.e., level of intellectual disability, psychiatric and neurological comorbidities), and to assess the presence of cognitive decline associated with dementia.

**Table 1:** Description of cognitive tests and informant-based questionnaires used for the study.

Test name	Primary ability assessed	Description	Outcomes
**Whisper Hearing Test (** Prescott et al. 1999 **)**	Hearing abilities	Participants are required to point to the image named by the researcher	Quietest voice tone heard (threshold = loud voice)
**Kaufman Brief Intelligence Test 2 (KBIT-2;** Kaufman 2004**)**	General cognitive and verbal abilities	Participants are required to identify correct answers in two verbal and one non verbal subtests	Verbal raw score (0–108); non verbal raw score (0–46); verbal IQ (40–160); non verbal IQ (40–132); Composite IQ (40–160)
**CAMCOG Verbal fluency (** Hon et al. 1999 **)**	Verbal abilities	Participants are asked to name as many animals as they can in 1 minute	Number of unique animals
**CANTAB Paired Associates Learning (PAL;** Robbins et al. 1994**)**	Memory	Participants observe and recall pattern locations	First trial memory score (0–26); number of stages completed (0–8)
**CANTAB Simple Reaction Time (SRT;** Robbins et al. 1994**)**	Processing speed/attention	Reaction time test requiring participants to press a button in response to a white square appearing	Mean response time; response time standard deviation; total number of correct responses (0–100)
**Tower of London (** Shallice 1982 **; adapted by** Strydom et al. 2007**)**	Executive functions	Participants move beads on a board to match presented configurations	Total score (0–10)
**Cambridge Examination for Mental Disorders of Older People with Down Syndrome and Others with Intellectual Disability (** Roth et al. 1986 **; CAMDEX-DS;** Ball et al. 2004**).**	Cognitive decline associated with dementia	Informant-based questionnaire related to cognitive decline in nine different domains	Everyday skills, memory and orientation, other cognitive skills, language, perception, praxis, executive functions, personality, and behavior (decline present/no decline)

General cognitive and verbal abilities were assessed using the Kaufman Brief Intelligence Test Second Edition (KBIT-2; Kaufman 2004) and the CAMCOG semantic verbal fluency test (Hon et al. 1999). The KBIT-2 is an IQ test that provides raw composite scores of verbal and non verbal general cognitive abilities, which can be converted to an age adjusted IQ score. For this study, we used both the age-adjusted IQ scores and the raw scores due to the high floor effect when raw scores are converted to IQ scores (Edgin et al. 2010; Startin et al. 2016). The CAMCOG semantic verbal fluency test is a measure of executive function with performance relating to vocabulary size and lexical access speed. Participants were asked to name as many animals as they could in 1 minute. The outcome was the number of unique animals named. Performance on the Tower of London task was used as an additional measure of executive functioning; in this task participants moved beads on a board to match presented configurations, with the outcome score based on the number of trials successfully completed and the number of moves made.

Memory abilities were measured through the Paired Associates Learning (PAL; Robbins et al. 1994) task, which is a measure of visuospatial short-term memory from the Cambridge Neuropsychological Test Automated Batteries (CANTAB). Participants are required to remember locations of an increasing number of patterns in progressive stages. The main outcomes from this test were the number of pattern locations correctly remembered on the first trial for each stage attempted and the number of stages completed. Finally, attention was assessed using the CANTAB Simple Reaction Time (SRT; Robbins et al. 1994) task. Participants are required to press a button as soon as they see a white box appear on the screen, with the main outcomes being the mean response time, the standard deviation for response times, and the total number of correct responses.

Finally, in participants aged 36 and above the presence of cognitive decline associated with dementia was measured using the Cambridge Examination for Mental Disorders of Older People with Down syndrome and Others with Intellectual Disability (CAMDEX-DS; Roth et al. 1986; Ball et al. 2004). The CAMDEX-DS is an informant-based questionnaire that assess the presence of decline in nine different domains (everyday skills, memory and orientation, general cognitive functioning, language, perception, praxis, executive functions, personality and behavior, and self-care).

### ERPs recording and MMN stimuli

Continuous EEG data were obtained using appropriately sized EGI HydroCel high-density sensor nets (containing 128 channel silver–silver chloride electrodes). Electrodes above and below the eyes recorded vertical electro-oculogram (VEOG), while those beside the outer canthus of each eye recorded horizontal electro-oculogram (HEOG). Electrode impedances were maintained below 50kΩ. During the recording, EEG signal was referenced to the vertex, using a 0.1–100 Hz bandpass filter, and then amplified using a gain of 10,000 and sampled at a rate of 250 Hz. Recordings were made using NetStation (Electrical Geodesics, Inc., Eugene, OR), while information regarding stimulus onset was sent via a parallel port using E-prime software (version 1.x).

During the EEG recording, participants were seated in a comfortable chair and were asked not to move during the recordings. A passive auditory oddball paradigm was employed, in which repetitive “Frequent” sounds were presented alongside two different types of rare “Deviant” sounds. The Frequent sound was a low-pitched “u” sound (U-low) and represented 70% of the stimuli. The first deviant tone was a “Pitch” deviant tone, where a high-pitched “u” sound (U-high) was played. The second deviant tone was a “Tone” deviant and consisted of a low pitched “i” sound (I-low). Each deviant tone represented 15% of the stimuli. Each tone had a duration of 100 ms, a rise and fall of 5 ms, and variable inter-stimulus intervals of 2–2.2 s. At the start of each block, 10 “U-low” sounds were presented to establish these as “Frequent.” Neural responses to these sounds were not recorded. There were 7 blocks containing 40 stimuli each, with a total of 42 stimuli for each deviant stimuli and 196 Frequent sounds. The speaker was set to 70 dB and placed in front of the computer screen midline. Participants were asked to focus on a fixation cross appearing on a computer monitor positioned 110 cm in front of them at eye level for the whole EEG recording time.

### E‌EG data pre-processing

Offline, EEG data were imported to EEGLAB toolbox 2021.0 (Delorme and Makeig 2004) running under Matlab R2021a (The MathWorks, Natick, MA), with the additional ERPLAB 8.20 plug-in (Delorme and Makeig 2004). EEG signal was digitally filtered using low-pass and high-pass filters set to 30 and 0.1 Hz, respectively. To reduce artifacts from the continuous EEG data (i.e. eye movements, eye blink, muscle contractions and movement) EEGLAB plugin *clean_rawdata()* including artifact subspace reconstruction (ASR) was applied to the data (Gabard-Durnam et al. 2018; Blum et al. 2019; Chang et al. 2019). The parameters used were flat line removal, 5 s; electrode correlation, 0.8; ASR, 20; window rejection, 0.5. As a result, the mean channel rejection rate was 6.5% (SD 2.6, range 2.5–13.7), while the mean data rejection rate was 2% (SD 2.9, range 0–14). The rejected channels were interpolated using EEGLAB's spline interpolation function and the signal was then re-referenced to the average electrode. Continuous data were segmented into 600-ms epochs from 100 ms pre-stimulus to 500 ms post stimulus onset and, successively, baseline corrected from −100 ms to 0 ms before the onset of the stimulus. Datasets with less than 11 epochs per condition were excluded from the analyses.

### M‌MN data extraction

**Fig. 1:**
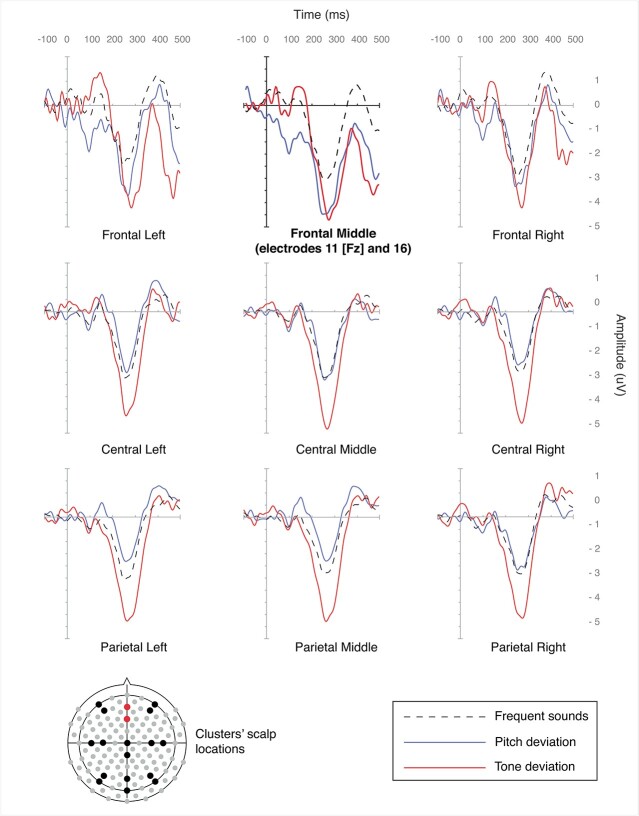
Grand average waveforms of the three stimulus types from different clusters across the scalp. Electrodes 11 and 16 (frontal middle scalp location) were used for the analyses.

Data from two fronto-midline electrodes (electrodes 11 [Fz] and 16; see Fig. 1) were extracted and used for the analyses. Several studies reported increased ERP latencies for the MMN in populations with DS even when no hearing impairments were observed (Lalo et al. 2005; César et al. 2010; Arisi et al. 2012; Kazan et al. 2016); therefore, we selected a 200-ms to 350-ms time window for the MMN amplitude and latency detection. For each participant, the MMN peak was defined as the most negative value between 200 and 350 ms and the MMN latency was computed accordingly. The Pitch deviant MMN was obtained by subtracting the ERP for the Frequent sounds from the ERP for the Pitch deviant stimuli, while the Tone deviant MMN was obtained by subtracting the ERP for the Frequent sounds from the ERP for the Tone deviant stimuli. Amplitudes and latencies from both Pitch and Tone deviant MMN were extracted for statistical analyses.

### Statistical analyses

All statistical analyses were performed using RStudio software (version 4.0.5; R Development Core Team 2021) and packages lme4 (Bates 2010), lmerTest (Kuznetsova and Brockhoff 2016), car (Fox and Weisberg 2018), and emmeans (Lenth et al. 2020). Linear mixed-effect models (LMMs) were used to assess whether the auditory response to the deviant sounds differed from the Frequent sounds. The type of stimuli (Frequent, Tone deviant, and Pitch deviant) was included as a fixed effect, and a random intercept was included for each subject to accommodate repeated measures. In addition, two models tested the impact of age and level of intellectual disability, respectively, on the MMN. The syntax of the models is reported in Table 2. Visual inspection of residual plots did not reveal deviations from homoscedasticity or normality. The significance of the fixed effects was evaluated using the *F* test with Satterthwaite approximation (Luke 2017). Post hoc pairwise comparisons were corrected with Tukey’s multiple comparison test. A *P* value < 0.05 was adopted for statistical significance. For significant interactions between a continuous variable (i.e. patients’ age) and a factor variable (i.e. type of stimuli), an estimated marginal means contrast was performed at the level of the 30th, 50th, and 70th percentile of the continuous variable.

**Table 2:** Model syntax (R software).

Model	Syntax
ERP Amplitude	Model_Amplitude = lmer(Amplitude − Type of stimuli + (1|ID), dataset)
ERP Latency	Model_Latency = lmer(Latency − Type of stimuli + (1|ID), dataset)
ERP Amplitude × age	Model_Age = lmer(Amplitude − Type of stimuli **×** Age + (1|ID), dataset)
ERP Amplitude × ID level	Model_IntellectualDisability = lmer(Amplitude − Type of stimuli ^*^ ID + (1|ID), dataset)

ERP amplitude or latency was the dependent variables in the models, whereas stimuli type (tone deviant, pitch deviant, and frequent), age, and level of intellectual disability (ID; mild, moderate, and severe) were the independent variables.

Correlations between mean amplitude and latency of both Pitch and Tone deviant MMN and neuropsychological scores were conducted using Spearman’s rank correlation coefficients. This exploratory approach enabled the examination of potential associations between the auditory response and cognitive function without a priori hypotheses. To minimize type I error likelihood arising from multiple correlations, the alpha significance level was set to *P <* 0.01.

## Results

### Sample description

**Fig. 2:**
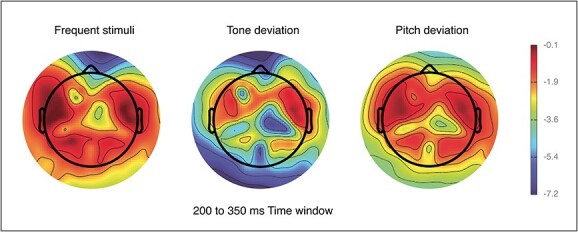
Grand average topographic map showing ERP components for frequent, tone deviant, and pitch deviant stimuli at 200–350 ms.

**Fig. 3:**
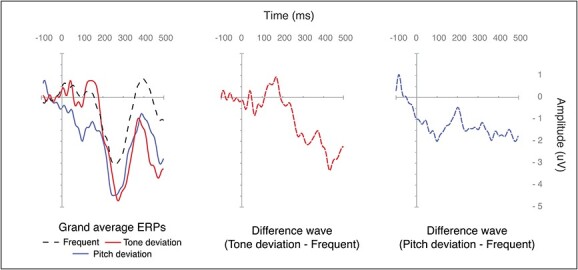
Grand average waveforms and difference waveforms for the three stimulus types at frontal middle scalp location. The panel on the left displays the grand average waveforms for frequent, tone deviant, and pitch deviant conditions at the frontal middle scalp location. The central panel depicts the difference waveform between the tone deviant and frequent conditions. The panel on the right shows the difference waveform between the pitch deviant and frequent conditions.

From a total of 47 datasets, 10 were excluded from the analyses as they presented with a low number of epochs after preprocessing (less than 11 epochs per conditions). Movement artifact represented the main source of noise in the EEG data. A visual check of the remaining EEG data revealed that some datasets did not present any ERP waveform. Therefore, a visual inspection approach was adopted for the exclusion of those datasets. Two independent raters blinded to the cognitive and demographic data evaluated all EEG data and datasets were removed if both raters concluded there was no ERP waveform present. As a result, EEG datasets from 10 individuals were excluded from the analysis. The final sample for analyses consisted of 27 individuals (*M* = 32.4 years, SD = 11.5; age range = 17–51; 14 females), of which 12 had mild intellectual disability, 14 moderate, and 1 severe. CAMDEX-DS data was available from 9 participants only (aged 36 and above) and, therefore, was not included in the analyses, but used for descriptive purposes only. In the final sample, 3 participants showed cognitive decline as measured by CAMDEX-DS, while none were diagnosed with dementia. The sample excluded from the analyses consisted of 20 individuals (*M* = 34.3 years, *SD* = 11.9; age range = 18–58; 12 females), 5 of which had mild intellectual disability, 11 moderate, and 4 severe. CAMDEX-DS data was available for 7 participants, 5 of which showed cognitive decline in at least one area of functioning (see Supplementary Table 1 for full details). The average ERP waveforms at different scalp positions are shown in Fig. 1, the topographic distribution is shown in Fig. 2, and the ERP difference waveforms at frontal middle scalp location is shown in Fig. 3.

### Linear mixed-effect models

**Fig. 4:**
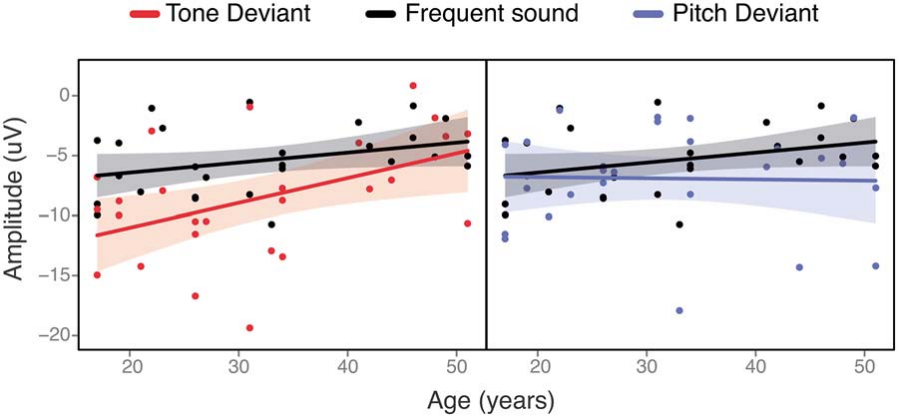
Age effect on the amplitude for the three different stimulus types. The plot shows the interaction between the ERP amplitude by participant age for the frequent sound, tone deviant, and pitch deviant stimuli. The difference between the waves for the tone deviant sound and frequent sound was statistically significant only in younger individuals with DS (contrasts at 26 and 31 years of age).

The model “ERP amplitude” investigating the ERP amplitude differences between the three stimuli showed a significant effect (see Table 3). The *F* test revealed significant differences between stimuli [*F*(2, 52) = 4.46, *P* = 0.01]. Post hoc contrasts showed significantly larger amplitudes for Tone deviant compared to Frequent sounds (−8.44 vs. −5.38, respectively; *P* = 0.01), but no significant difference between Pitch deviant and Frequent sounds (−7.43 vs. −5.38, respectively; *P* = 0.13) or Tone deviant and Pitch deviant stimuli (−8.44 vs. −7.43, respectively; *P* = 0.60). The model “ERP latency” investigating ERP latency differences between the three stimuli showed no significant effect [*F*(2, 52) = 0.26, *P* = 0.79]. A significant type of stimuli × age interaction was found [*F*(2, 50) = 4.98, *P* = 0.01] in the “ERP amplitude × age” model. Contrasts were conducted at the level of 30th, 50th, and 70th percentile of the covariable age, corresponding to 26, 31, and 41 years, respectively. Post hoc tests revealed a significant difference between the Tone deviant and Frequent sounds only at 26 years (−9.76 vs. −5.91, respectively; *P* = 0.003) and 31 years (−8.73 vs. −5.50, respectively; *P* = 0.005) but was absent at 41 years (−6.66 vs. −4.66, respectively; *P* = 0.2) as shown in Fig. 4. Finally, the model “ERP Amplitude × ID level” investigating potential association between ERPs amplitude and level of intellectual disability showed no significant effect [*F*(4, 52) = 0.19, *P* = 0.94].

**Table 3:** ERP amplitude and latency (*n* = 27).

Condition	Amplitude (μV)	SD amplitude (μV)	Mean latency (ms)	SD latency (ms)
Frequent	−5.38	2.80	258	34.84
Tone deviant	−8.43	5.02	258	35.41
Pitch deviant	−7.42	5.01	254	32.50

μV = microvolt; ms = millisecond

### Spearman’s rank correlation coefficients

**Fig. 5:**
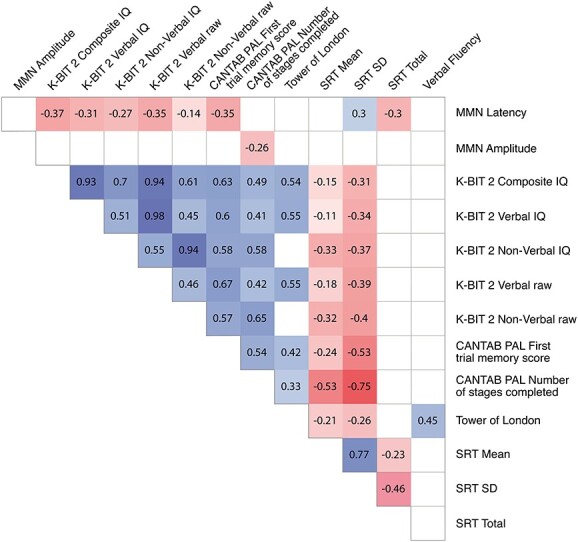
Spearman’s correlation between MMN parameters and cognitive task scores (*P* < 0.01). MMN = mismatch negativity (i.e. difference between tone deviant and frequent sound); K-BIT 2 = Kaufman brief intelligence test 2; K-BIT 2 verbal raw = Kaufman brief intelligence test 2—verbal raw score; K-BIT 2 non verbal raw = Kaufman brief intelligence test 2—non verbal raw score; CANTAB PAL = paired associates learning; SRT mean = simple reaction time—mean response time; SRT SD = simple reaction time—response time standard deviation; SRT total = simple reaction time—number of correct responses.

The amplitude of the Tone deviant MMN (i.e. the difference between Tone deviant and Frequent sounds) significantly negatively correlated with the number of PAL stages completed (*r* = −0.26, *P <* 0.01). Finally, Tone deviant MMN latency significantly negatively correlated with the KBIT-2 composite IQ score (*r* = −0.37, *P* < 0.0001), KBIT-2 verbal IQ score (*r* = −0.31, *P <* 0.0001), KBIT-2 verbal raw score (*r* = −0.35, *P* < 0.0001), KBIT-2 non verbal IQ score (*r* = −0.27, *P <* 0.001), PAL first trial memory score (*r* = −0.35, *P* < 0.0001), SRT total score (*r* = −0.30, *P* < 0.01), and significantly positively correlated with the SRT standard deviation score (*r* = 0.30, *P* < 0.001), as shown in Fig. 5.

## Discussion

The present study aimed to characterize the relationship between MMN amplitude and latency and cognitive abilities in people with DS. An auditory oddball paradigm in which the Frequent sound was alternated with deviations in pitch and tone was employed. Potential associations between MMN parameters, age, verbal abilities, memory, attention, and executive functioning were tested.

### M‌MN and age

There was a significant interaction between ERP amplitude for the three types of stimuli and age, whereby the contrast between Frequent sound and Tone deviations generated a significant difference in ERPs amplitudes only in young adults with DS (up to 31 years of age), while the contrast did not reach statistical significance in older adults. These results are in keeping with Avancini et al. (2020), where age inversely predicted MMN amplitude in adults with DS, but not in typically developing individuals. This may be due to difficulties in the maintenance of the sensory memory trace for Frequent sounds in older individuals with DS, leading to a reduced detection of deviant sounds. Supporting this hypothesis, we found that the increased Tone deviant MMN (hereinafter referred as “MMN”) amplitude correlated with better performance in the CANTAB PAL, a computerized task measuring memory and learning abilities (Barnett et al. 2015). Furthermore, a recent cross-sectional examination of cognitive markers of AD in our larger study of 312 participants with DS found that memory and attention were the most sensitive cognitive markers of AD-related decline, and started to show changes from 40 years of age (Startin et al. 2019).

In recent years, the MMN has been interpreted within the predictive coding framework. Predictive coding postulates that sensorial perception is an active process in which the brain, in order to infer the most probable (hidden) cause of incoming sensory data, generates internal models of the outside world. These models are constrained by the statistical regularities of the external environment and are used to compute top–down predictions (i.e., “priors”) aimed at “explaining away” the incoming sensory signal in a Bayesian fashion (Friston 2005; Clark 2013). The bottom–up sensory stream is thought to be processed against the priors and only the difference between what has been predicted and the actual sensory input (i.e., “prediction error”) is fed-forward up the neural hierarchy, to update the models. Within this theorization, the MMN is considered to reflect the prediction errors as it is elicited by deviant stimuli presented with low probability (violating, therefore, the predictions; Garrido et al. 2009). The difference we found in our sample, which consists of an attenuation of the MMN as a function of age, may therefore indicate the presence of impairment within regularity violation detection processes in older people with DS. Specifically, it can be hypothesized that the expected precision (or inverse variance) attributed to the incoming sensory input was low in older participants, potentially resulting in the suppression of the prediction errors (i.e. MMN) and the increase of the model’s posterior probability (Friston 2009).

Another important aspect that requires consideration is Aβ-amyloid deposition associated with AD, which is commonly observed in brains of people with DS by the age of 40 (Hartley et al. 2014; Jennings et al. 2015; Annus et al. 2016) and the concomitant decline in memory (Hartley et al. 2017). Interestingly, only older participants from our sample did not exhibit MMN. It could be hypothesized that Aβ-amyloid accumulation in temporal brain regions may have impacted the ability to maintain sensory memory traces for the Frequent sounds in older participants. Impaired formation of the sensory memory trace will affect the activity of the frontal (i.e., comparator) MMN generator, causing a reduction in the ERP response amplitude. Taken together, this evidence suggests that the absence of MMN in our older adult sample may reflect difficulties in maintaining sensory memory traces and this may be due to AD-associated neuroanatomical and cognitive decline. However, the relation between MMN and Aβ-amyloid deposition proposed here is speculative and further research is required to support this conclusion.

### M‌MN latency and verbal abilities

In the present study, the ERPs latency was longer than canonical parameters reported in the literature (Näätänen et al. 2007). This is in keeping with previous studies in populations with DS, wherein the MMN latency was longer regardless of hearing threshold (Diaz and Zurron 1995; Lalo et al. 2005; César et al. 2010; Arisi et al. 2012). Interestingly, we found an association between the MMN latency and KBIT-2 verbal IQ as well as KBIT-2 verbal raw score. Specifically, longer MMN latencies were associated with reduced verbal IQ and reduced verbal raw scores. Therefore, delays in the discrimination of regularity violation, as indexed by prolonged MMN latencies, may underly impairments in verbal abilities, which is one of the main hallmarks of cognition in DS (Grieco et al. 2015). Supporting this hypothesis, an association between MMN latency and verbal abilities has frequently been reported in ASD. Roberts et al. (2011) studied the effects of concomitant language impairments in ASD using magnetic mismatch field (MMF), the magnetic equivalent of the MMN. The authors divided the children with ASD in two groups, one with and one without concomitant language impairments, and found that the MMF latency was significantly longer in the former group. A receiver operator characteristic analysis of the mean MMF latency indicated a sensitivity of 82.4% and a specificity of 71.2% of the peak latency for the presence of language impairments. The authors concluded that the MMF latency may reflect a neurobiological basis and clinical biomarker for language impairments in ASD. In a subsequent study, Roberts et al. (2012), replicated the same results in a population without ASD. The authors compared a group of young adults with specific language impairments with a group of age-matched typically developing individuals. The results showed a 92% contribution of the MMF latency in differentiating the two groups. These findings are supported by other studies showing that a longer MMF/MMN latency predicts language impairments in ASD, people with specific language impairments (Cardy et al. 2005; Kasai et al. 2005; Ingalhalikar et al. 2014; Port et al. 2015; Matsuzaki et al. 2019; Chen et al. 2020), and in a population with auditory processing disorder (Rocha-Muniz et al. 2015). Therefore, the prolonged MMN latency observed in our sample, as well as in previous studies of the MMN in DS, may be interpreted as a neurophysiological indicator of speech signal processing impairments in DS.

Finally, an important consideration is whether the MMN delays observed in people with DS primarily indexes impairments in speech-sound discrimination, or whether the delay reflects a more general widespread cognitive impairment as well as a reduction in processing speed. The speech signal is composed by harmonical elements that change rapidly in regard to their frequency (i.e., formant transitions). In this scenario, the ability to accurately process speech relies on the capacity to identify and interpret rapid and transient changes in the acoustic signal. For instance, the identification of syllables is determined by rapid shifts in the distribution of spectral energy between phonemic segments. When considering that the acoustic properties of speech sounds are encoded at all levels of the auditory system, even small delays in these processes could profoundly compromise downstream comprehension mechanisms (Griffiths 1999; Nicol and Kraus 2004). Therefore, it would be reasonable to assume that delays in speech-sound processing may have an impact on subsequent processing stages, (e.g., semantic processing), with a consequent detrimental effect that is not limited solely to an individual’s verbal abilities. To this end, Näätänen et al. (2011) proposed that the MMN attenuation/delay may index (beside affected central auditory processing) cognitive decline irrespective of specific etiology and symptomatology among different neuropsychiatric conditions. Accordingly, we also observed associations between the MMN latency and KBIT-2 composite IQ as well as CANTAB PAL, where increases in the MMN latencies corresponded to poorer task performance. Therefore, the delays in speech-sound processing in DS, as indexed by the MMN latency, may have a cascading effect leading to the impairment of a broad array of higher-order cognitive processes.

### Neurodevelopmental and neurodegenerative mechanisms underlying cortical responses slowdown

What remains unknown is whether the observed delay in the MMN response observed in people with DS is driven by neurodevelopmental or neurodegenerative mechanisms. As previously mentioned, slowdown of cortical responses is a common feature in people with DS, being frequently reported for ERP components other than MMN, such as the N100, P200, and P300. While some studies have described an association with age and AD diagnosis (Blackwood et al. 1988; Muir et al. 1988), others have reported slowdown of cortical responses regardless of the age of participants (Callner 1978; Dustman and Callner 1979; Diaz and Zurron 1995). As the latency of ERP components may increase in relation to the time taken by an individual to perceive a stimulus or to compute a process (Hall 2007; Näätänen et al. 2012), the prolonged MMN latency observed in our sample may therefore be caused by a reduced neural reactivity in people with DS. However, a reduction of cortical reactivity could be the end product of either neurodevelopmental or neurodegenerative mechanisms, or a combination of these.

When considering neurodevelopment in people with DS, different mechanisms could potentially explain our findings. From a biological point of view, abnormalities in monoamine neurotransmitter functions (Lake et al. 1979), reduced electrical permeability of neural membranes and dendritic spine numbers (Lott and Dierssen 2010), as well as changes in synaptic morphology (Scott et al. 1983; Lott and Dierssen 2010) could underlie the reduced cerebral reactivity in people with DS (Arisi et al. 2012). As a consequence, the slowdown of information processing in people with DS may cause a slowness of MMN-related cognitive processes such as attention, comparison, coding, evaluation, and classification (Lincoln et al. 1985; Lalo et al. 2005). Finally, brain development in people with DS is characterized by volume reduction affecting predominantly brain regions deemed to be responsible for MMN generation (i.e., temporal and frontal cortices). This may cause, for instance, an impairment within information integration processes between the auditory association areas and other cortical and subcortical brain areas, further contributing to the observed delay in MMN responses (César et al. 2010).

An alternative and complementary hypothesis about the potential neurobiological basis of cortical response slowdown in adults with DS is related to AD neurodegenerative mechanisms affecting the integrity of the brain’s white matter in this population. The fronto-temporal brain network is a collection of brain areas responsible for auditory and language processing, as well as for MMN generation (Catani and Jones 2005; Garrido et al. 2007; Garrido et al. 2008; Oestreich et al. 2019). This brain network is physically connected through the auditory white matter pathway of the arcuate fasciculus and inferior fronto-occipital fasciculus (Catani and Jones 2005; Martino et al. 2010), and through the auditory interhemispheric pathway of the corpus callosum (Wigand et al. 2015). Interestingly, several diffusion-based MRI studies in people with DS have consistently shown microstructural atrophy affecting all these white matter structures (Powell et al. 2014; Gunbey et al. 2017; Romano et al. 2018). It is assumed that the ERPs latencies are, at least in part, a reflection of the degree of white matter myelination (Thomas and Crow 1994; for a review see e.g., von Siebenthal et al. 1994). Therefore, white matter integrity loss may underlie the prolonged MMN and ERP latency observed in people with DS.

The Retrogenesis model is a theory that describes the time progression of AD related to neurodegeneration affecting the brain’s white matter. This model states that white matter degeneration in AD reflects the reverse of the myelogenesis developmental order (Reisberg et al. 1999), whereby the tracts that myelinate later in development (such as the long association fibers) are the first to be affected in AD (Bartzokis 2004). Interestingly, all the main long association fibers connecting the frontal lobes with the rest of the brain seem to be affected in the population with DS (see Saini et al. 2022 for a review). In this context, structural problems (e.g., demyelination) affecting frontal lobe connectivity may lead to impairments in executive function processes that are essential for MMN generation (e.g., attention, comparison, and inhibitory control). In line with this, we found a correlation between MMN latency and the standard deviation of the SRT, a task measuring response time. Specifically, longer MMN latencies were associated with higher response time standard deviations. Within brain processing speed literature, response time standard deviation is considered a measure of intra-individual performance variability. Intra-individual performance variability (or inconsistency) is defined as within-person performance fluctuations across trials or multiple testing sessions (Rabbitt et al. 2001), and is deemed as a cognitive marker of central nervous system integrity, as well as an index of the efficiency with which executive control processes are implemented (for a discussion see Macdonald et al. 2006). Within the typically developing population, intra-individual performance variability increases with age (Hultsch et al. 2002) and is associated with changes in brain morphology, such as white matter loss. Specifically, it has been linked to frontal lobe white matter hyperintensities in elderly individuals from the general population (Bunce et al. 2007) and to corpus callosum size in individuals with mild cognitive impairment (Anstey et al. 2007). Therefore, the integrity loss of the white matter tracts connecting the frontal lobes with the rest of the brain in people with DS may underlie impairments in attentional and executive function processes that are essential for the MMN generation. Consequently, interferences within the frontal comparator mechanisms may be experienced when incoming stimuli are processed, leading to extended latencies of the MMN and late ERP components. In turn, delays in speech-discrimination and/or in regularity violation detection processes may lead to global and detrimental effects on cognition.

Finally, it has been demonstrated that pre-attentive auditory processes responsible for MMN generation are modulated by the cholinergic system (Pekkonen et al. 2001; Pekkonen et al. 2005). Degeneration of cholinergic neurons is a common feature of AD and pharmacological therapies commonly used in this pathology aim to compensate reduced cholinergic activity (Seltzer 2006; Musial et al. 2007; Hampel et al. 2018). Impairments affecting the cholinergic system have also been described in DS (Granholm et al. 2000; Isacson et al. 2002; Fodale et al. 2006) and are likely caused by AD-related neurodegenerative processes (Kish et al. 1989; Isacson et al. 2002). Therefore, degeneration of the cholinergic system could be one of the neurodegenerative mechanisms explaining the prolonged MMN latencies observed in DS (Pekkonen et al. 2007).

In conclusion, based on the present observations, it could be hypothesized that the extended MMN latencies observed in people with DS may be driven by neurodevelopmental mechanisms such as histological and monoaminergic anomalies, which may be further impacted by AD-related neurodegenerative mechanisms such as white matter demyelination and cholinergic degeneration during adulthood.

### Strengths, limitations, and future directions

This present study contains noteworthy strengths. Firstly, compared to previous studies of the MMN in DS, in which only young adults were assessed, the present study included adults with a wide age range (17–51 years). This allowed us to explore age-related differences in the electrophysiological features of the MMN. Furthermore, we employed an extensive cognitive battery testing a wide range of cognitive domains. One limitation of our study is the use of the Whisper Hearing Test to assess participants' hearing status, rather than pure tone audiometry. Whilst we excluded participants with significant hearing loss based on this screening tool, it is possible that some participants may have had mild hearing loss that went undetected. Considering the importance of preserved auditory function for the MMN generation, future studies could benefit from using more sensitive hearing assessments, such as pure tone audiometry, to ensure that participants have normal hearing or only mild hearing loss.

The absence of a control group meant we were unable to explore potential differences in the electrophysiological features of the MMN between individuals with DS and typically developing individuals; however, there is already an existing literature on controlled studies (Lalo et al. 2005; Pekkonen et al. 2007; César et al. 2010; Arisi et al. 2012), and this was not directly relevant to our aims.

Additionally, whilst the correlational analyses provide insight into the relationship between MMN characteristics and cognitive function, it should be noted that they were conducted in an exploratory manner without correction for multiple comparisons, which may increase the risk of Type I errors. The oddball interstimulus interval was set at a short rate, meaning we could not explore the effect of more varied interstimulus intervals on memory trace maintenance. Finally, given the high rate of movement artifact observed in some of our participants and the consequent epochs removal, future studies may benefit from employing an oddball task with only one deviant stimulus and increased number of repetitions.

## Conclusions

In conclusion, the present study shows that the MMN was elicited only in young adults with DS. This may be due to difficulties in the maintenance of sensory memory traces for the Frequent sounds, potentially caused by AD-related neurodegeneration in older individuals with DS. Furthermore, the MMN tended to have longer latencies when compared to canonical parameters reported in the literature. The prolonged MMN latencies appear to be associated with memory, verbal IQ, and general IQ, suggesting that delays in speech-sound processing may underlie a more global detrimental effect on cognition in this population that precedes the development of AD; though this might be further worsened by AD pathology. Cortical response slowdown has also been reported by previous studies of the MMN in people with DS and may result from neurodevelopmental processes such as histological and monoaminergic anomalies. However, AD-related neurodegenerative processes affecting the brain’s white matter myelination may potentially exacerbate the reduced cortical reactivity in this population. Specifically, demyelination of frontal lobe white matter may impair regularity violation detection and attentional switch processing, both of which are essential for the MMN generation.

## Supplementary Material

Supplementary_Table_1_bhad233

## Data Availability

Data are available upon reasonable request. Requests for access to data from the study should be addressed to fedal.saini@kcl.ac.uk. All proposals requesting data access will need to specify planned uses with approval of the study team before data release.
