# Supplementary material for: The mismatch negativity as an index of cognitive abilities in adults with Down syndrome
Source: Cereb Cortex. 2023 Jul 3;33(16):9639–51. doi: 10.1093/cercor/bhad233 (PMC10431748; doi:10.1093/cercor/bhad233)
Supplement: Supplementary_Table_1_bhad233 [file supplementary_table_1_bhad233.docx]

**Supplementary materials**

**Supplementary Table 1:** Participant characteristics and assessment details

| ID | Study inclusion | Age | Sex | ID Level | Whisper hearing test | Assessments gap (days) | CAMDEX-DS decline |
| --- | --- | --- | --- | --- | --- | --- | --- |
| Subj01 | Yes | 17 | M | Moderate | Whispered | 69 | -- |
| Subj02 | Yes | 17 | F | Moderate | Whispered | 42 | -- |
| Subj03 | Yes | 17 | F | Mild | Whispered | 183 | -- |
| Subj04 | No | 18 | M | Severe | Whispered | 17 | -- |
| Subj05 | Yes | 19 | M | Moderate | Whispered | 75 | -- |
| Subj06 | Yes | 19 | M | Moderate | Whispered | 244 | -- |
| Subj07 | No | 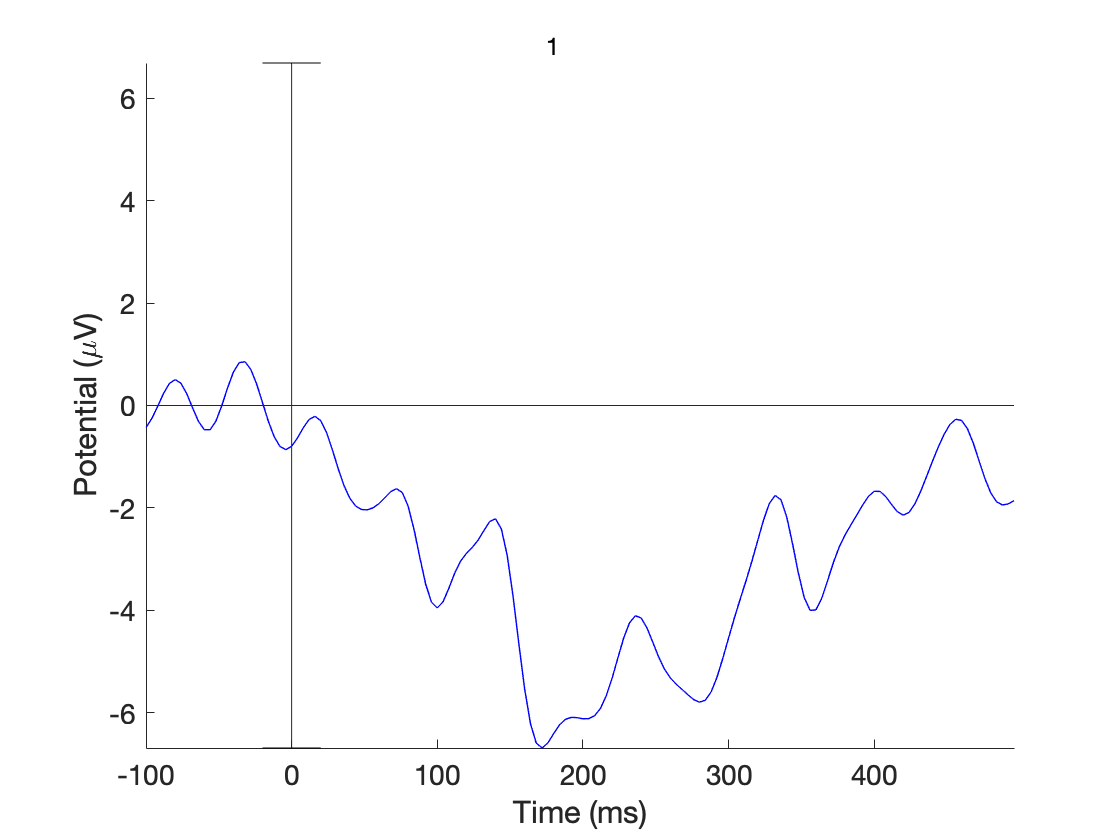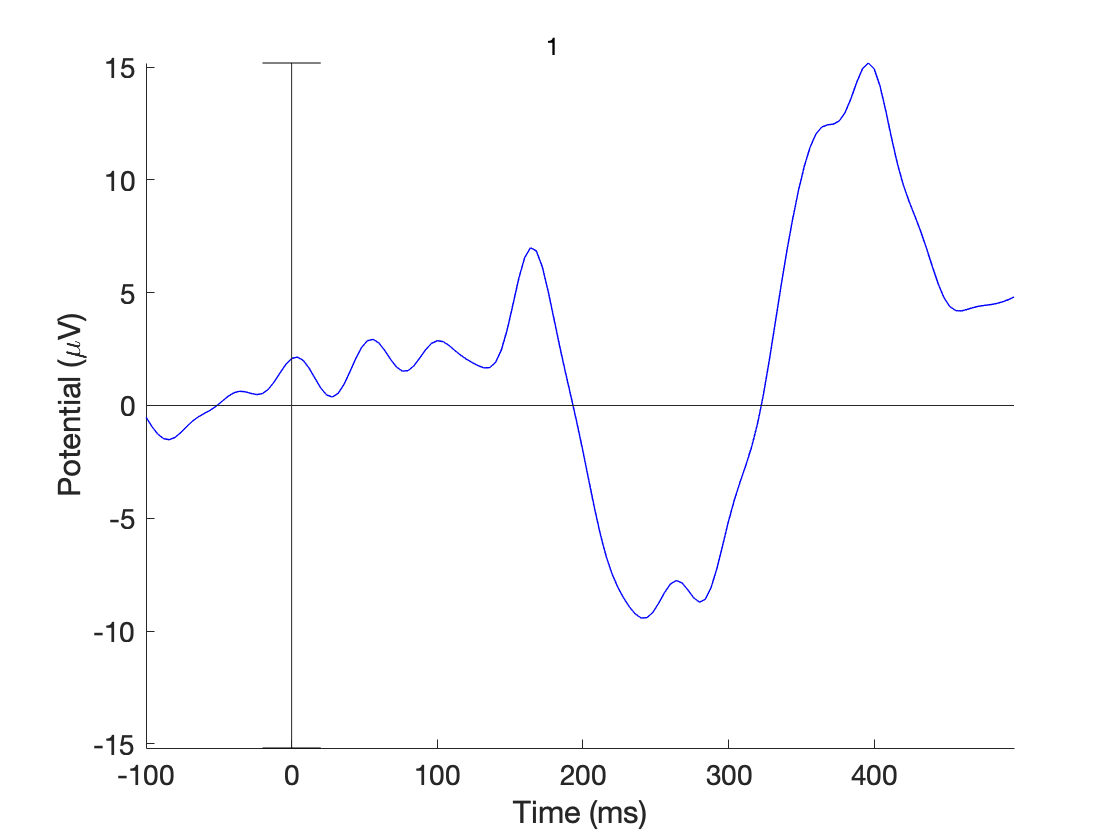20 | M | Moderate | Whispered | 244 | -- |
| Subj08 | No | 21 | M | Severe | Whispered | 162 | -- |
| Subj09 | Yes | 21 | M | Moderate | Whispered | 556 | -- |
| Subj10 | Yes | 22 | M | Severe | Whispered | 171 | -- |
| Subj11 | Yes | 23 | M | Moderate | Whispered | 48 | -- |
| Subj12 | No | 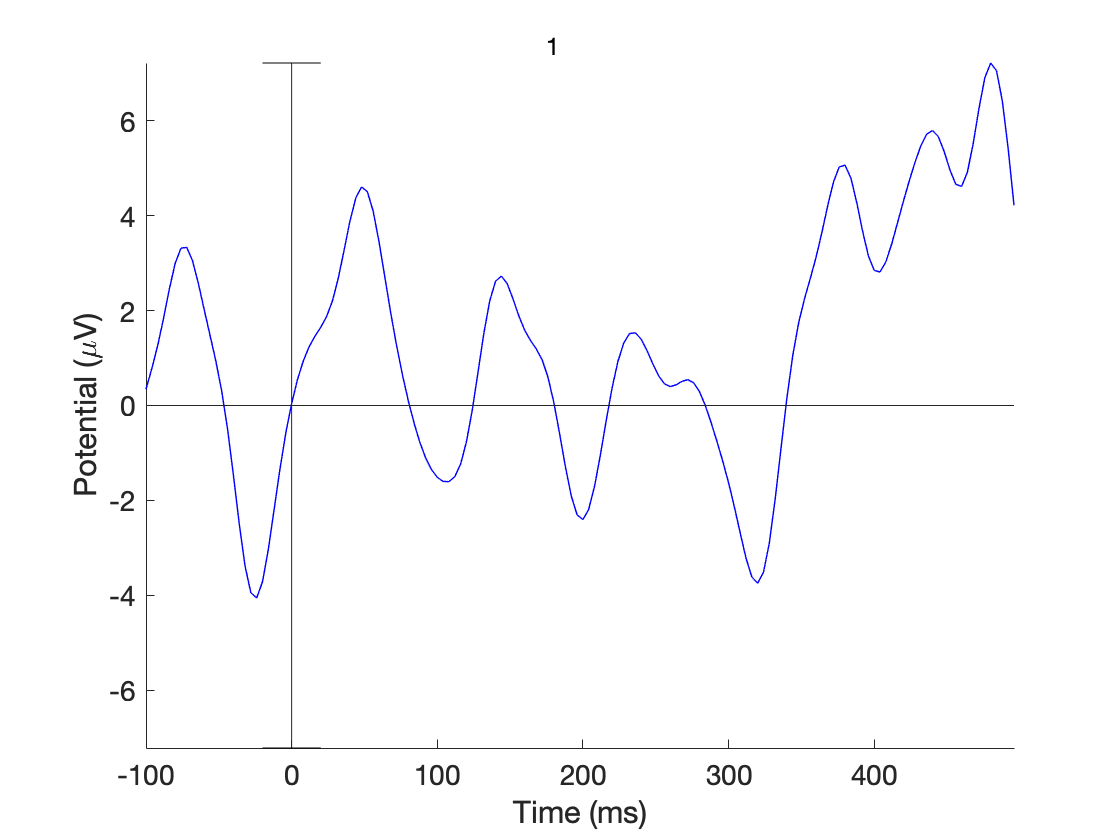25 | F | Mild | Conversational | 169 | -- |
| Subj13 | No | 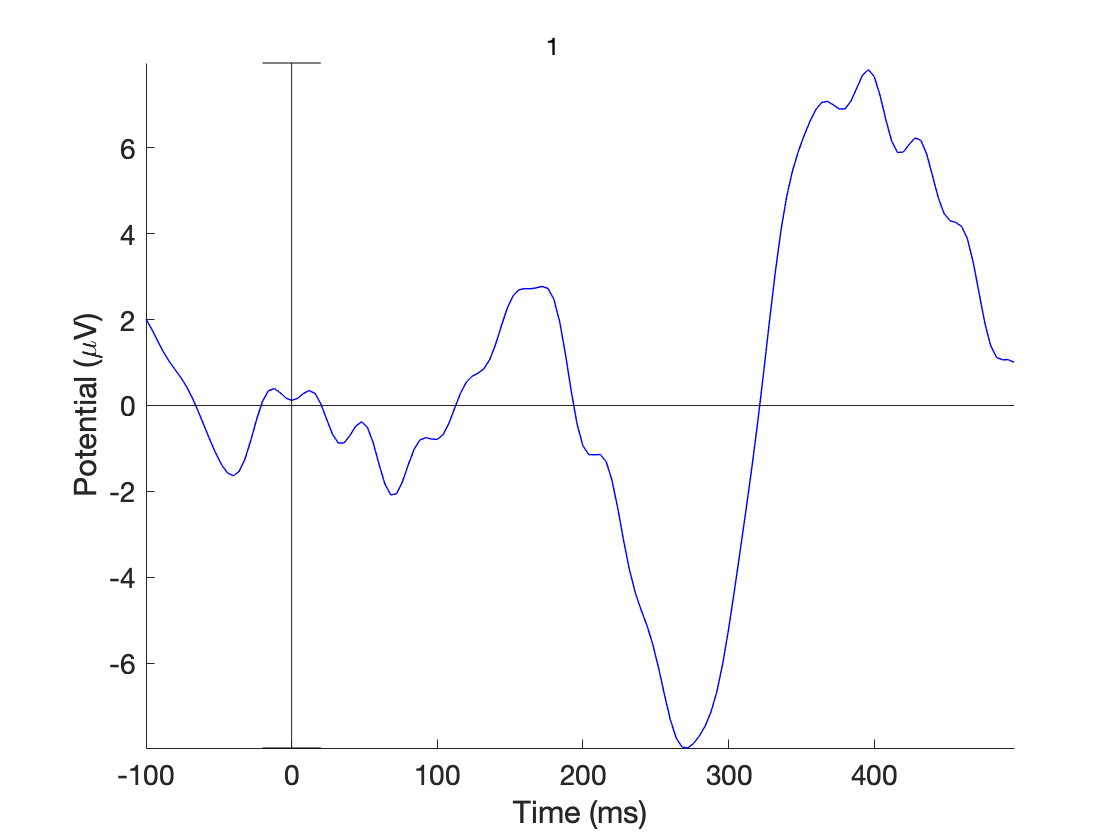25 | F | Moderate | Whispered | 212 | -- |
| Subj14 | Yes | 26 | F | Mild | Whispered | 182 | -- |
| Subj15 | Yes | 26 | F | Moderate | Whispered | 254 | -- |
| Subj16 | No | 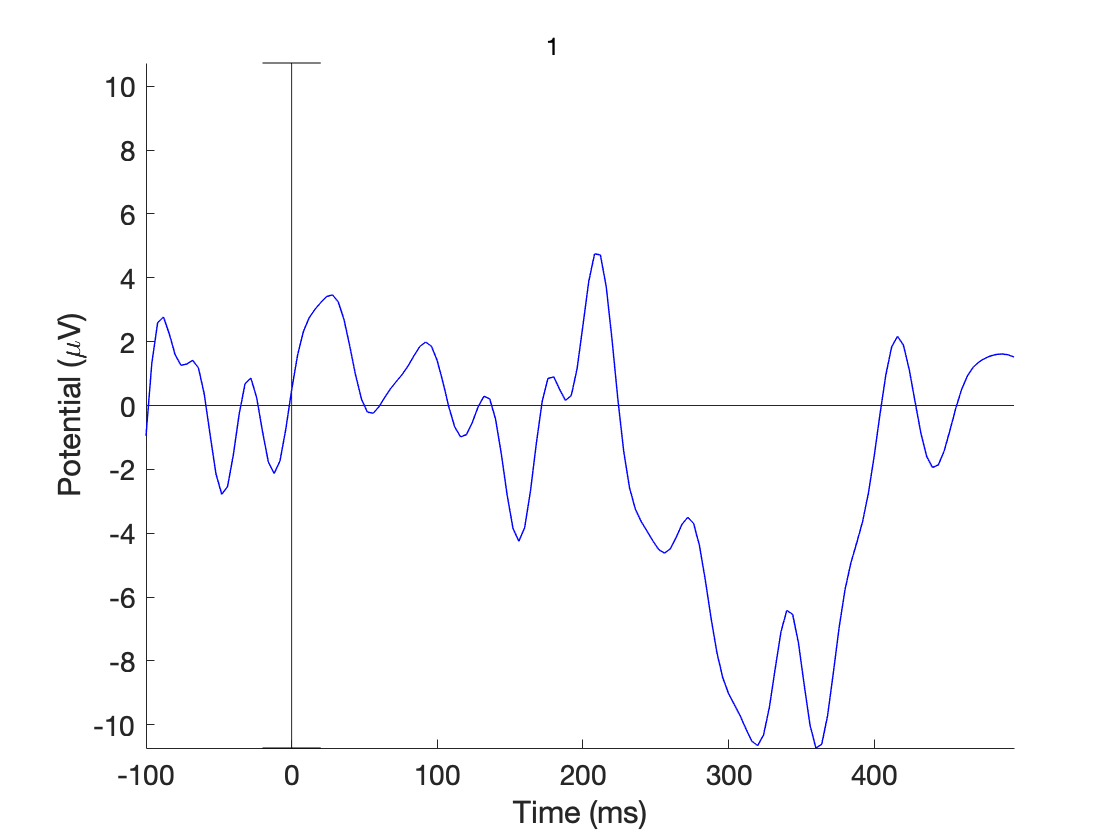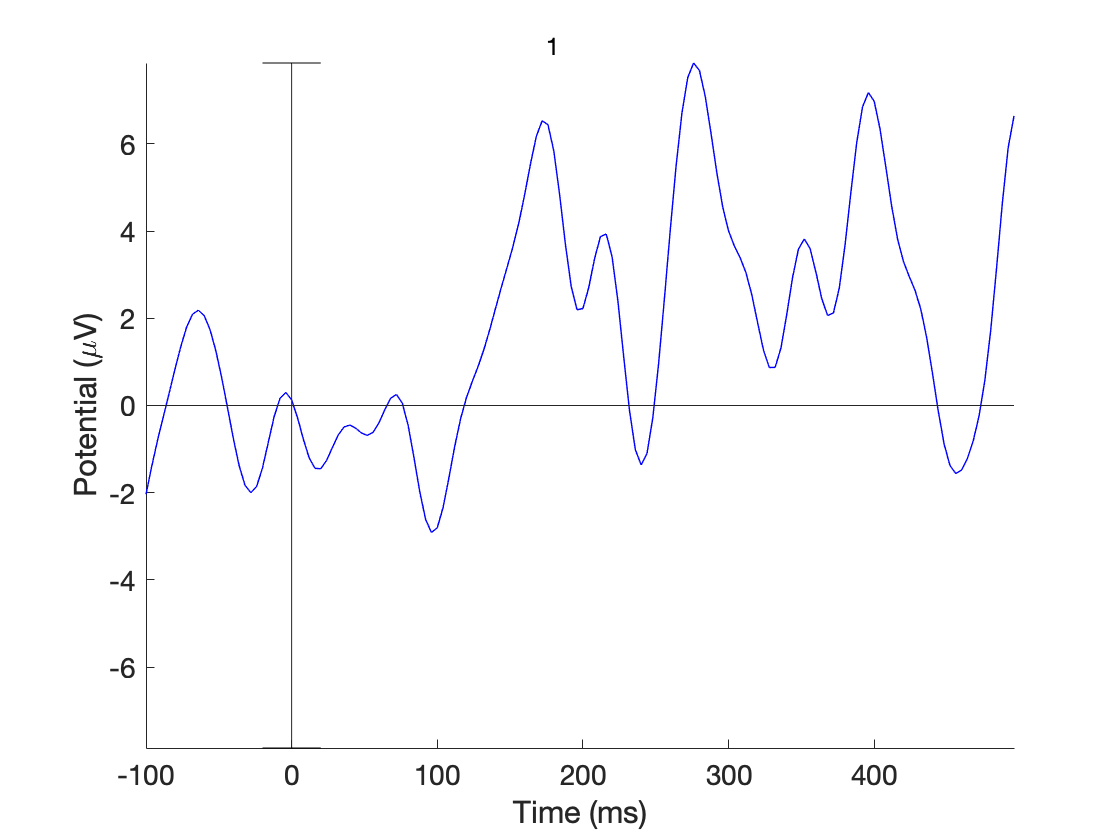26 | M | Mild | Whispered | 83 | -- |
| Subj17 | No | 26 | M | Moderate | Whispered | 26 | -- |
| Subj18 | Yes | 26 | F | Mild | Whispered | 43 | -- |
| Subj19 | Yes | 27 | M | Mild | Whispered | 237 | -- |
| Subj20 | No | 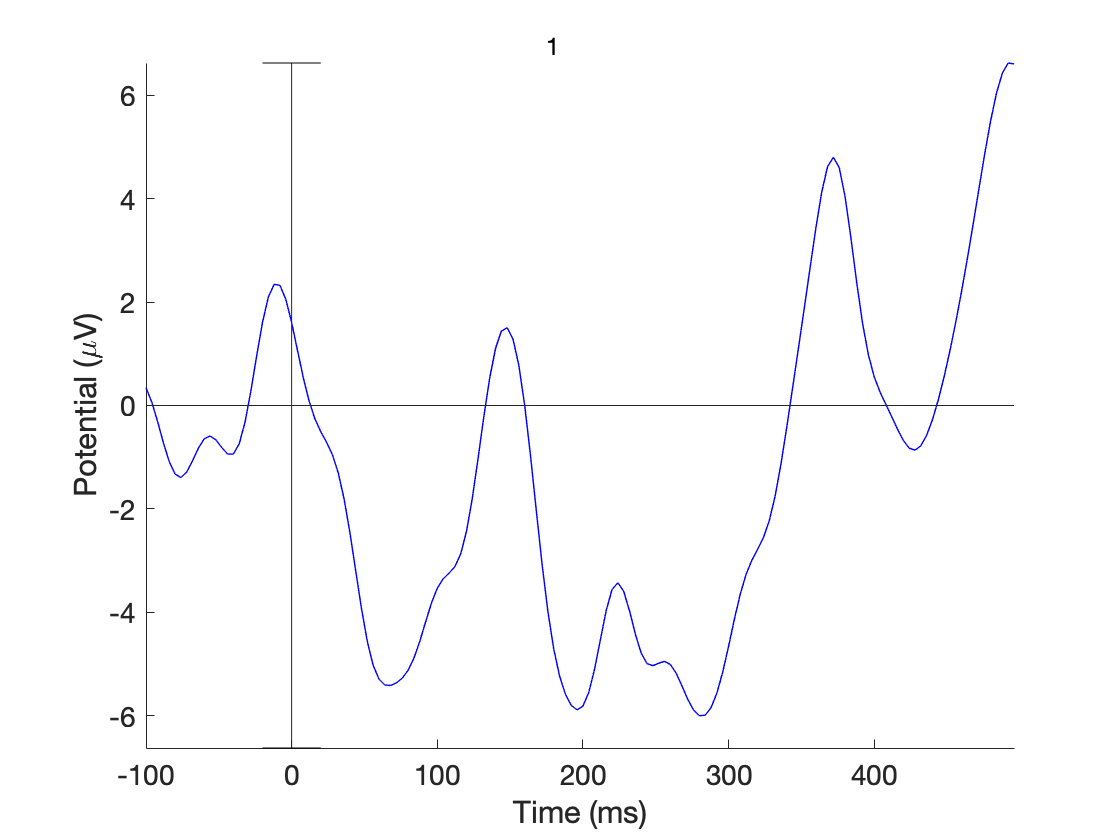31 | F | Mild | Whispered | 203 | -- |
| Subj21 | Yes | 31 | F | Mild | Whispered | 714 | -- |
| Subj22 | Yes | 31 | F | Moderate | Whispered | 106 | -- |
| Subj23 | No | 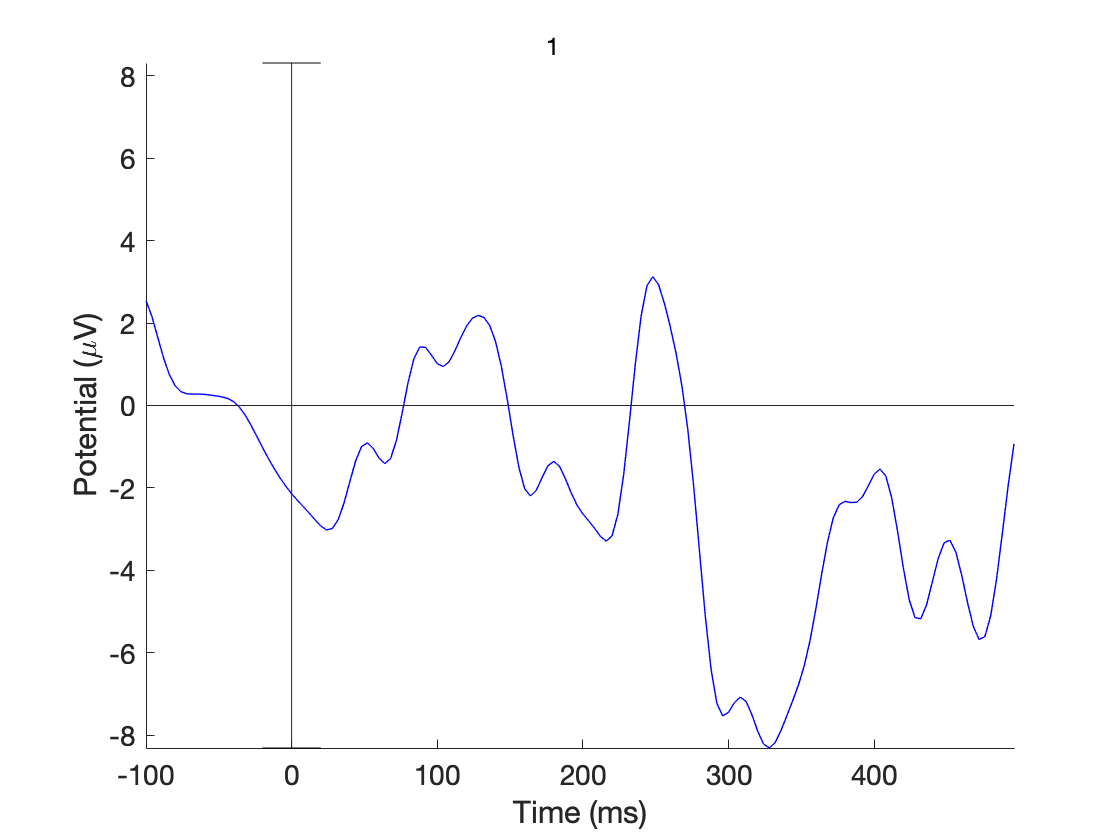32 | M | Moderate | Whispered | 112 | -- |
| Subj24 | No | 32 | F | Moderate | Whispered | 70 | -- |
| Subj25 | No | 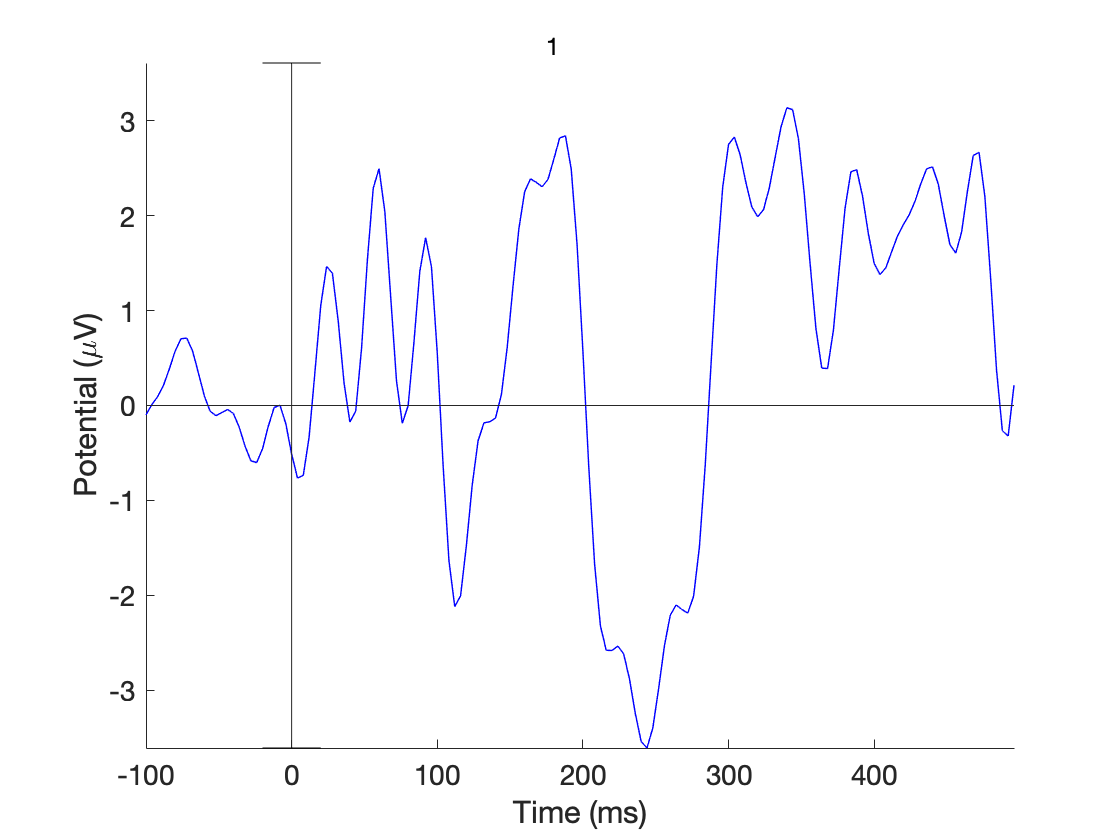32 | M | Moderate | Whispered | 240 | -- |
| Subj26 | Yes | 33 | F | Mild | Whispered | 85 | -- |
| Subj27 | Yes | 34 | F | Moderate | Whispered | 91 | -- |
| Subj28 | Yes | 34 | M | Mild | Whispered | 89 | -- |
| Subj29 | No | 34 | F | Mild | Whispered | 177 | -- |
| Subj30 | Yes | 34 | M | Moderate | Whispered | 145 | -- |
| Subj31 | No | 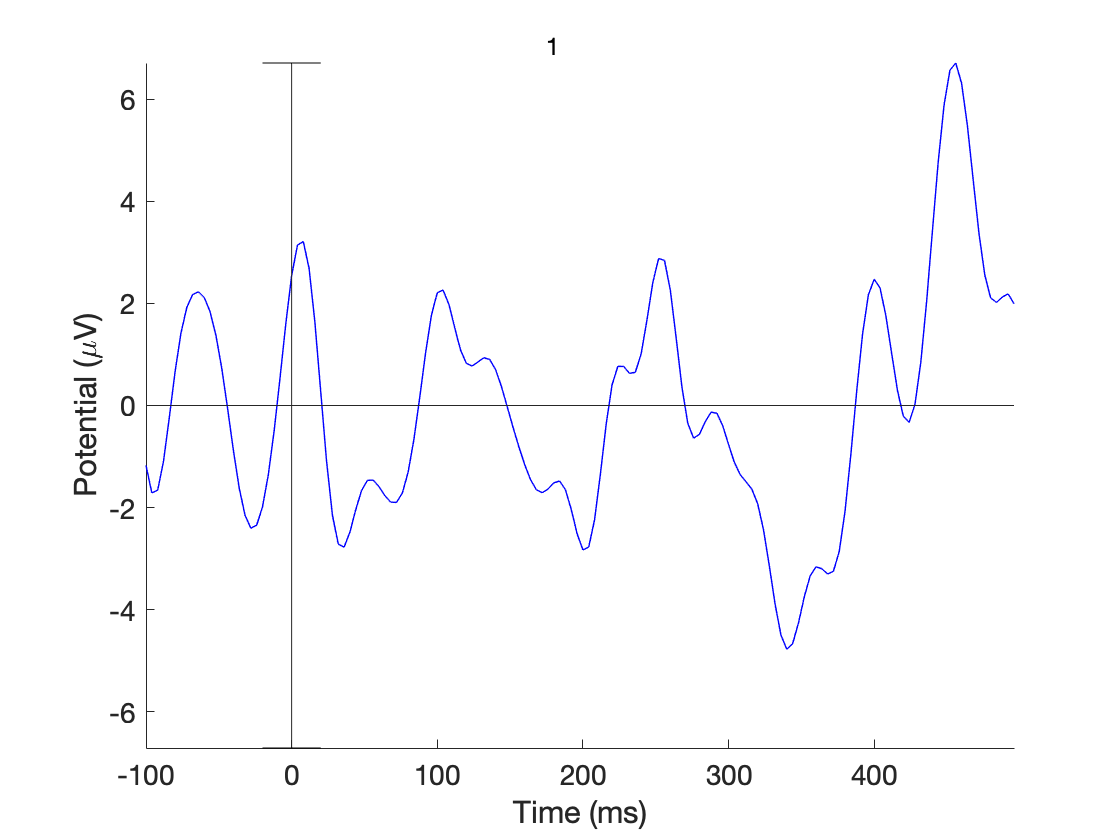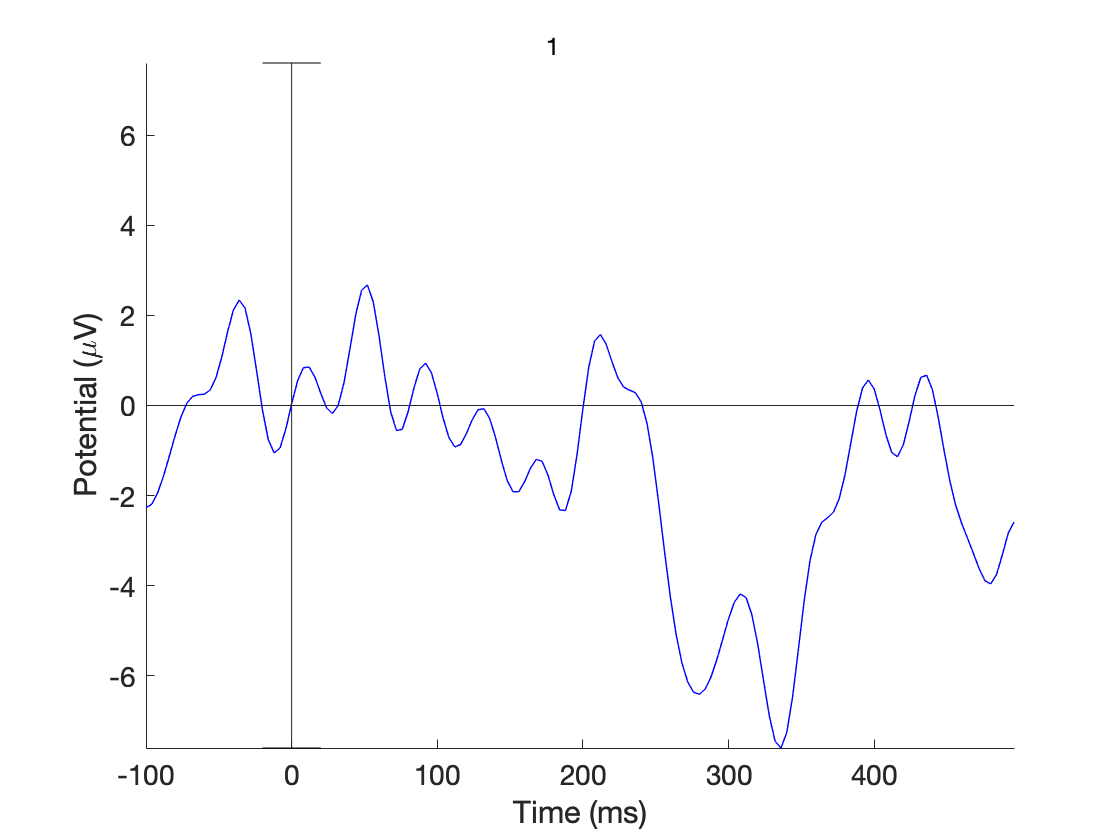35 | F | Moderate | Whispered | 162 | -- |
| Subj32 | No | 37 | F | Mild | Whispered | 155 | No |
| Subj33 | No | 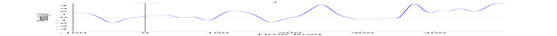37 | F | Moderate | Whispered | 28 | Yes |
| Subj34 | No | 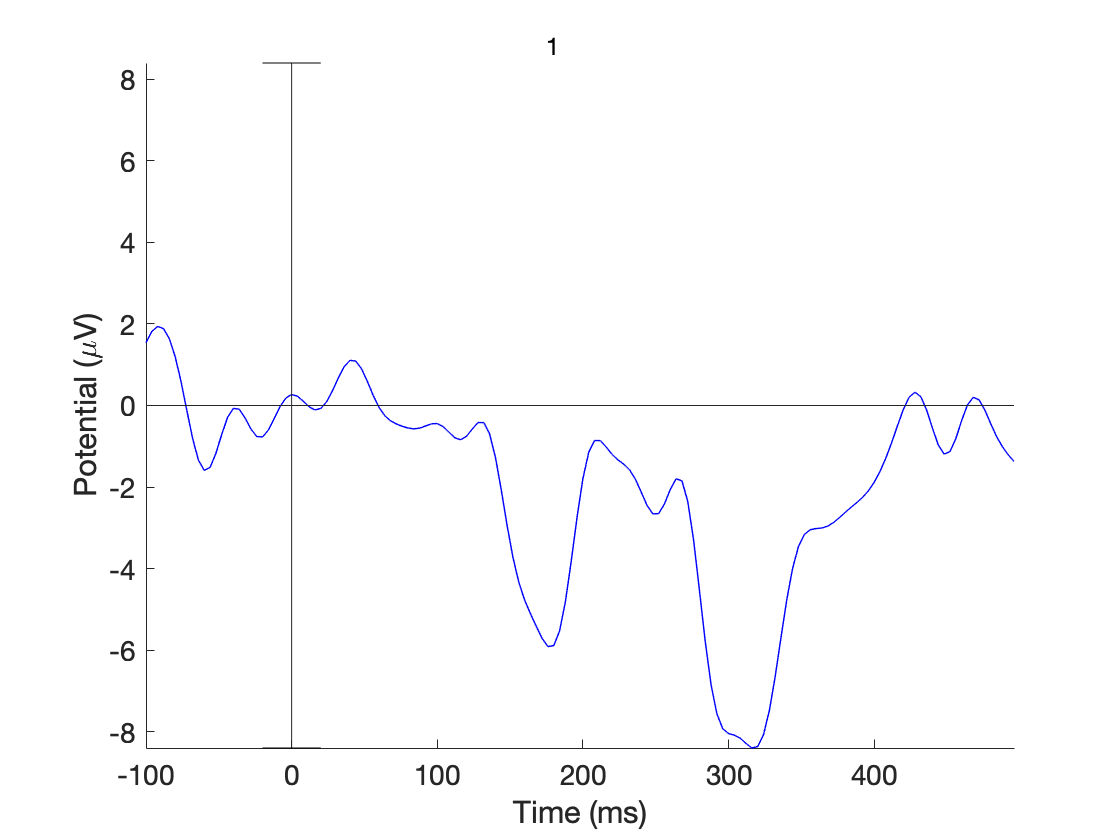39 | F | Severe | Whispered | 139 | Yes |
| Subj35 | Yes | 41 | F | Mild | Whispered | 172 | No |
| Subj36 | Yes | 42 | M | Moderate | Whispered | 24 | yes |
| Subj37 | Yes | 44 | F | Mild | Conversational | 0 | No |
| Subj38 | No | 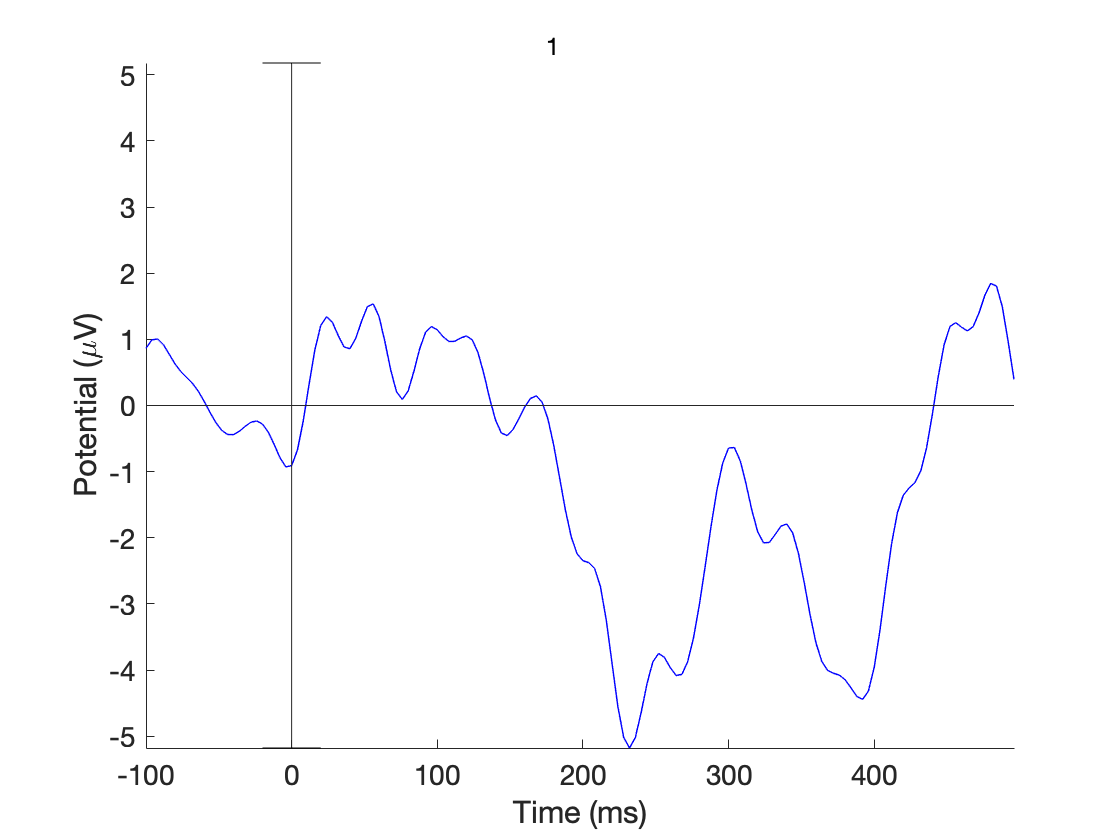45 | M | Moderate | Whispered | 151 | No |
| Subj39 | Yes | 46 | F | Moderate | Whispered | 59 | No |
| Subj40 | Yes | 46 | M | Mild | Whispered | 63 | No |
| Subj41 | Yes | 48 | F | Mild | Whispered | 77 | Yes |
| Subj42 | Yes | 49 | F | Moderate | Whispered | 135 | No |
| Subj43 | Yes | 51 | M | Mild | Whispered | 80 | No |
| Subj44 | Yes | 51 | M | Moderate | Whispered | 61 | No |
| Subj45 | No | 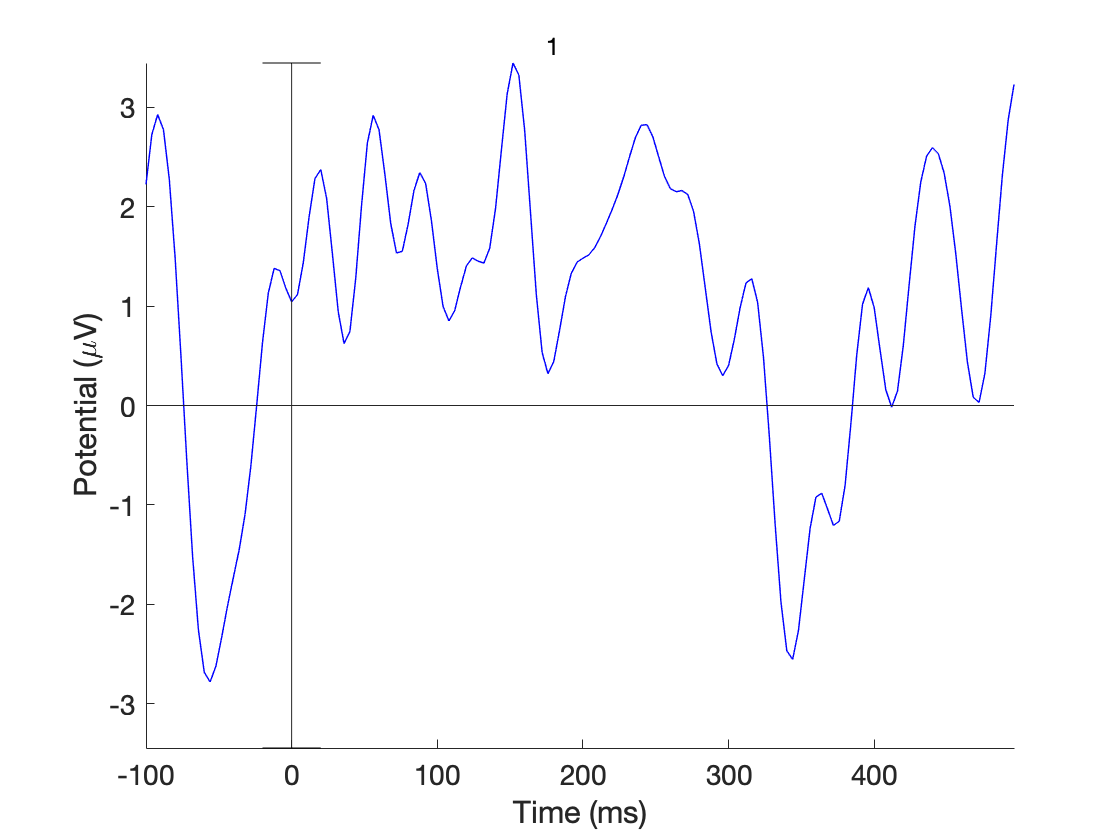56 | F | Moderate | Whispered | 99 | Yes |
| Subj46 | No | 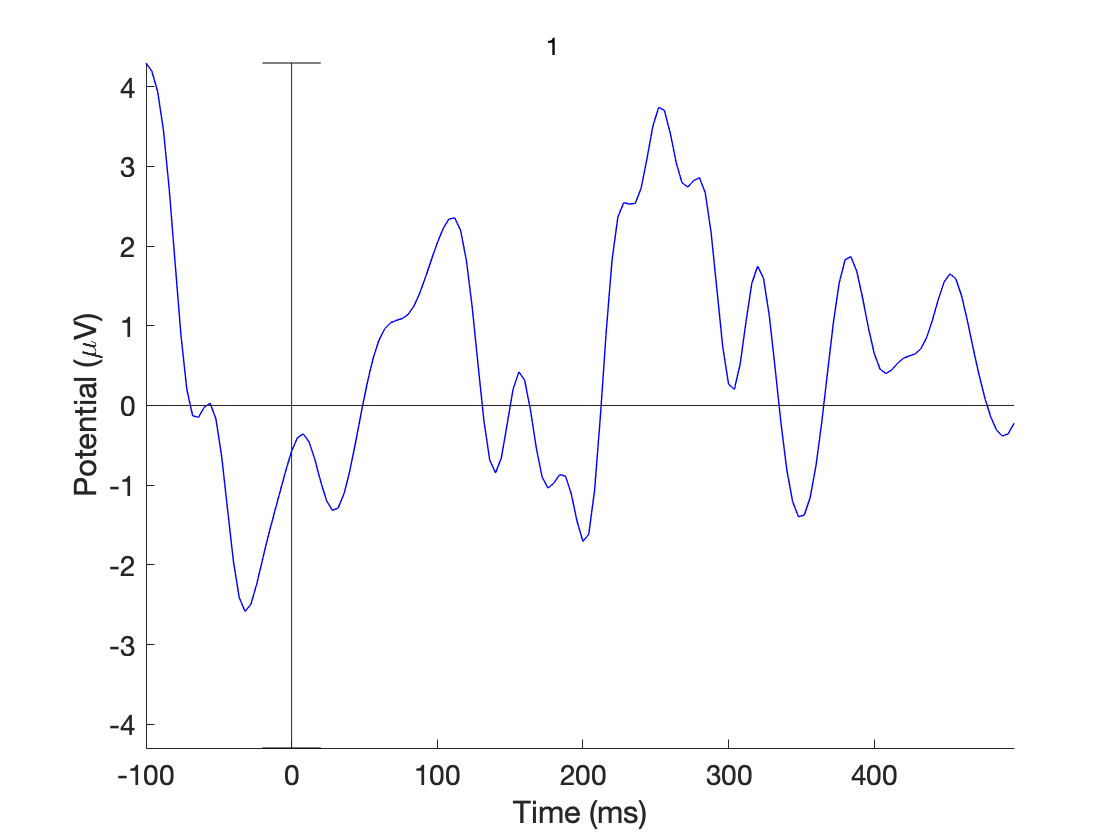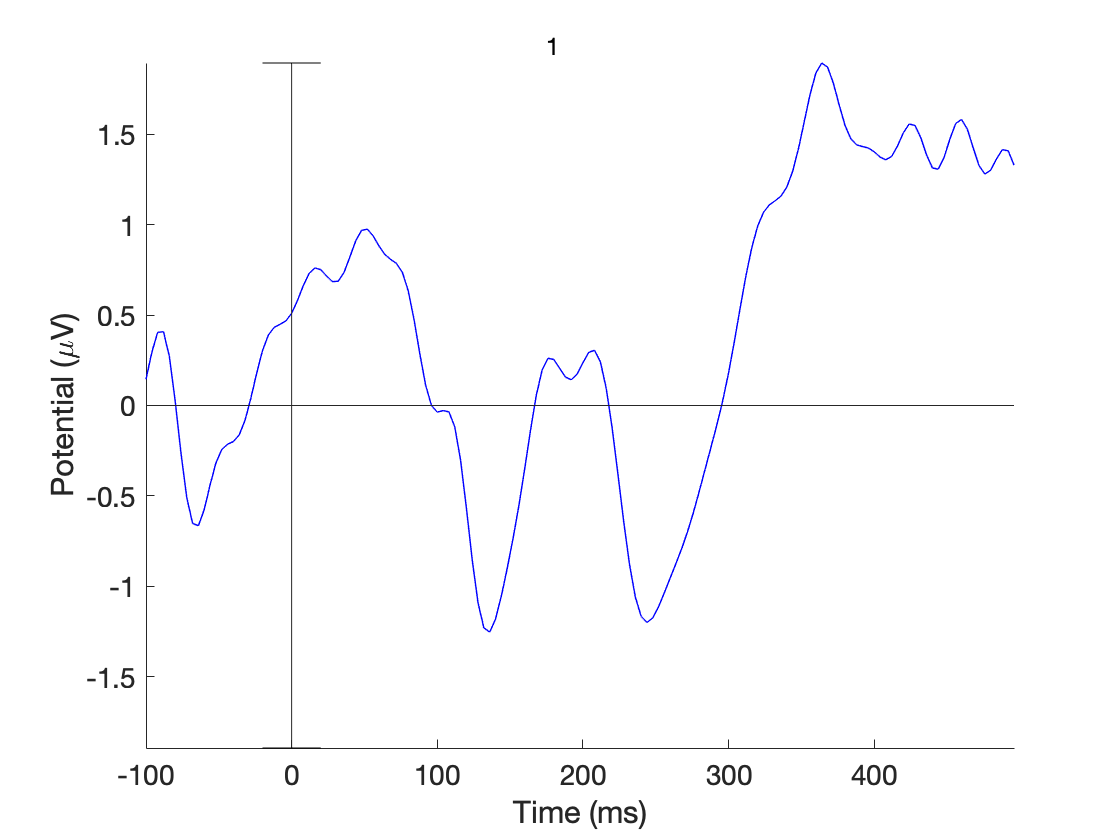58 | F | Moderate | Whispered | 129 | Yes |
| Subj47 | No | 58 | F | Severe | Whispered | 29 | Yes |

Study inclusion = participant inclusion (yes/no) based on data quality after EEG data pre-processing; ID level = Intellectual disability level; Assessments gap (days) = time gap in between cognitive assessment and EEG recording; CAMDEX-DS decline = presence of decline as measured with the Cambridge Examination for Mental Disorders of Older People with Down's Syndrome and Others with Intellectual Disabilities
